# The magic of mushrooms: psilocybin influences behavior in the mangrove rivulus fish, *Kryptolebias marmoratus*

**DOI:** 10.3389/fnbeh.2026.1767175

**Published:** 2026-05-07

**Authors:** Dayna Forsyth, Nicoletta Faraone, Simon G. Lamarre, Suzanne Currie

**Affiliations:** 1 Department of Biology, Acadia University, Wolfville, NS, Canada; 2 Department of Chemistry, Acadia University, Wolfville, NS, Canada; 3 Département de Biologie, Université de Moncton, Moncton, NB, Canada; 4 Department of Biology, University of British Columbia, Kelowna, BC, Canada

**Keywords:** activity, aggression, mangrove rivulus, psilocin, psilocybin, social behavior

## Abstract

Non-human models, including fish, are increasingly important for investigating how pharmacological agents such as hallucinogens influence behavior, physiology, and cellular processes. These models help to reveal underlying mechanisms and to support assessments of toxicological impact, efficacy, and safety. In this study, we used isogenic lineages of the amphibious mangrove rivulus (*Kryptolebias marmoratus*), an emerging model fish known for high activity and socially dynamic interactions. This species often display aggression towards conspecifics making it well-suited to study behavioral effects of low doses of the psychoactive compound, psilocybin. We determined whether psilocybin could induce calming effects and reduce social aggression and activity. We socially stimulated fish using pairs of size-matched fish from different isogenic lineages and compared baseline social behavior following a waterborne dose of psilocybin. Waterborne psilocybin treatment resulted in a significant decrease in activity levels and in the frequency of swimming bursts (an aggressive behavior) towards a conspecific fish from a different lineage, with modest alterations on other behaviors. Our results also revealed considerable intraspecific variation in the behavioral response of these homozygous fish, suggesting the effects of psilocybin were largely independent of genotype. This study demonstrates that psilocybin reduces aggression and activity in an emerging fish model, adding to the evidence supporting its potential as a therapeutic agent for future clinical translation.

## Introduction

Psilocybin is a naturally occurring psychoactive compound found in over 200 mushroom species, primarily within the *Psilocybe* genus (Van Court et al., 2022; Nichols, 2020; Plazas and Faraone, 2023). Highlighted for its favorable safety profile for humans compared to other hallucinogens (Gable, 1993, 2004; Hendricks et al., 2015), it shows increasing therapeutic potential for mental health disorders where conventional drugs have limitations (Madsen et al., 2019; Schindler et al., 2015; Castellanos et al., 2020). Psilocybin (pro-drug) dephosphorylates by alkaline phosphatases and esterase to its active form, psilocin, mainly in the liver (Donovan et al., 2021; Ling et al., 2022). Psilocin structurally resembles serotonin and crosses the blood-brain barrier, allowing it to bind to serotonin (5-HT) receptors, particularly the 5-HT_2A_ receptor in the mammalian brain (Braun et al., 2024; de Veen et al., 2017). These interactions influence human behavior and emotions, including aggression, appetite, mood, and memory. Although the exact molecular mechanisms remain unclear, a single dose of psilocybin has been associated with lasting mood improvements (Donovan et al., 2021). Psilocybin and psilocin have relatively short half-lives of approximately 160 and 50 min, respectively, in humans, and a 30–40 min half-life of psilocin in the plasma of pigs and under 1 h in mice (Hasler et al., 1997; Martin et al., 2013; Donovan et al., 2021; Thomann et al., 2024). Despite these short half-lives, they exert long-term behavioral effects, yet we know little about their therapeutic potential and mechanism of action. Non-human models help to reveal underlying mechanisms and to support assessments of toxicological impact, efficacy, and safety, all essential for studying the behavioral, physiological, and cellular consequences of psychedelic compounds (Syed et al., 2023).

Non-human models are commonly used in drug-screening experiments, providing robust results that can be later translated to humans. While there is recent evidence suggesting psilocybin influences the behavior of pigs (Donovan et al., 2021), mice (Zhuk et al., 2015; Zhao et al., 2024; Woodburn et al., 2024), and fishes (Abramson et al., 1963; Tombari et al., 2023; Braun et al., 2024), studies remain limited. Fish are valuable experimental models due to their genetic and physiological homology with humans, ease of lab maintenance, and cost-effectiveness (Shin and Fishman, 2002; Adeyinka et al., 2025). Early work showed psilocybin induced surface-floating behaviors in Siamese fighting fish (*Betta splendens)* (Abramson et al., 1963). Psilocybin research declined sharply after the Controlled Substances Act of 1970, remaining dormant for decades (Ortiz and Preuss, 2024). Since its reemergence, the limited research has primarily used larval zebrafish (*Danio rerio*) to investigate the neuro-behavioral effects of psilocybin (Braun et al., 2024; Tombari et al., 2023). Recent larval zebrafish studies comparing the effects of several hallucinogenic drugs found that psilocybin had one of the lowest potencies, with no observed behavioral alterations or teratological effects (Tombari et al., 2023). This minimal influence may result from psilocybin’s relatively selective agonist activity at the 5-HT_2A_ receptor and/or its short half-life, leading to rapid clearance from the body (Tombari et al., 2023). Conversely, Braun et al. (2024) observed that acute psilocybin exposure (2.5 μM for 4 h) in larval zebrafish enhanced spontaneous exploration and exerted an anti-stress effect by reversing stress-induced behavioral changes. This anti-stress effect is likely mediated by the suppression of serotonergic neurons, suggesting a conserved psilocybin mechanism among vertebrates (Braun et al., 2024; Adeyinka et al., 2025). These latter findings suggest that psilocybin may mitigate the physiological stress state, but a significant knowledge gap remains regarding its effects on a wider range of behaviors.

There is some preclinical evidence in humans revealing therapeutic properties of psilocybin in conditions that often impair social behavior such as depression and anxiety (Johnson and Griffiths, 2017; Back et al., 2024). Fish present as ideal models to understand potential effects of psilocybin on social behaviors. As one of the most speciose vertebrate groups, they exhibit an astonishing range of social organization, from vast oceanic aggregations and large schools to small groups of territorial and often aggressive fish (Ward et al., 2020). As discussed above, the limited psilocybin research on fishes suggests both stimulatory and anxiolytic effects on behavior. These responses are particularly relevant given that social interactions are crucial for establishing group dynamics, with aggressive behavior often playing a key role in competing for resources, securing mates, and defending territory (Larson et al., 2006). The amphibious mangrove rivulus fish *(Kryptolebias marmoratus)* is a highly relevant neuro-behavioral model, given its socially dynamic and naturally aggressive nature (Edenbrow and Croft, 2012a,b). The mangrove rivulus is one of two self-fertilizing hermaphroditic fish, allowing us to study genetic and environmental interactions, or nature versus nurture (Kelley et al., 2016). Through several generations of self-fertilization in laboratory populations, individuals within a lineage are genetically homozygous or isogenic, with each lineage being genetically distinct from the others (Kelley et al., 2016; Tatarenkov et al., 2015). We capitalized on this unique mode of reproduction to isolate and control for the genotype component of any observed experimental response. Mangrove rivulus are notably active fish (Pronko et al., 2013), and these activity levels are influenced by social experience and external stressors (Budaev et al., 1999; Currie and Tattersall, 2018; Melanson et al., 2023).

Given the complex link between sociality, aggression, and neurobiology, the mangrove rivulus provides a compelling model for understanding the behavioral effects of psilocybin (Adeyinka et al., 2025). Our goal was to determine how psilocybin, known for its therapeutic and calming properties, influences the aggression and activity levels of mangrove rivulus while controlling for genetic variation. Based on these therapeutic properties, we predicted that a single dose, administered via water immersion to isogenic mangrove rivulus in dyadic pairs would reduce both aggression and overall activity levels.

## Materials and methods

### Experimental animals

We housed *K. marmoratus* fish in a breeding colony at the Animal Care Facility at Acadia University, Wolfville, Nova Scotia, Canada. We used three distinct lineages: DAN originally from Dangriga, Belize (2006), and H9 and H11 originally from the Bay Islands, Honduras (1996) (Tatarenkov et al., 2010). These lineages have been bred in the laboratory population for over 45 generations, as mangrove rivulus can produce approximately three generations per year (Tatarenkov et al., 2010; Martin et al., 2021). We performed experiments on the DAN lineage (focal fish) with the H9 lineage as stimulus fish for behavioral experiments; we used the H11 lineage for the LC-MS analysis. Individuals were all drug- and experimentally-naïve adult hermaphrodites (older than 3 months). Fish were housed individually in 120 mL plastic cups with approximately 80 mL of 15 ppt synthetic seawater (Instant Ocean, J&L Aquatics, Burnaby, BC, Canada, and reverse osmosis water). The cups were maintained under controlled conditions with a 12L:12D photoperiod and a relative humidity of 30% at 24°C ± 2°C. We fed live *Artemia* nauplii (Brine Shrimp Direct, Ogden, UT, United States) four times weekly with a supplemental frozen bloodworm (Hikari Bio-Pure purchased from Pet Valu, New Minas, NS, CA) feeding once per week. Water changes and cleaning were completed every 9 days. All experimental trials were approved by the Acadia University Animal Care Committee as per the guidelines set by the Canadian Council on Animal Care (#05-19, #07-21, and #07-24).

### Behavioral effects of psilocybin following water immersion

#### Pharmacological treatment

Psilocybin was provided by Halucenex Inc., Windsor, Nova Scotia, Canada. Psilocybin is water-soluble, and we chose the initial concentration range and treatment time based on previous zebrafish studies done on LSD, MDMA and mescaline, which have similar serotonergic agonist properties (Grossman et al., 2010; Stewart et al., 2011; Kyzar et al., 2012). We conducted preliminary trials with three waterborne doses: 500, 1,000, and 3,000 μg/L which fall within the established effective concentrations for related psychoactive compounds in fish models (e.g., Grossman et al., 2010; Stewart et al., 2011; Kyzar et al., 2012) and determined that 3,000 μg/L induced the strongest behavioral response. To ensure the physiological relevance of this dose, this concentration was validated in the subsequent LC-MS analysis, which confirmed that the resultant internal drug concentrations corresponded to low–moderate therapeutic dose range in human studies (Holze et al., 2023; Fang et al., 2024).

We administered psilocybin to the focal fish by adding it to 15 ppt saltwater in an opaque square glass tank (8.0 L × 8.0 W × 8.0 H cm; thickness 0.5 cm) and placing each mangrove rivulus in this tank for 20 min. A new waterborne psilocybin water bath was prepared for each individual fish. This exposure time was based on previous zebrafish hallucinogenic studies (Grossman et al., 2010; Pham et al., 2012). Psilocybin has a short half-life *in vivo*, leading to a sustained duration of action per dose when compared to a similar hallucinogen, LSD (Abramson et al., 1963). Therefore, we opted for an acute exposure time of 20 min, and given the quick onset of action, we assessed the response 15 min after treatment.

#### Experimental protocol

We analyzed fish behavior through blinded video analysis. We conducted all experiments from 10 a.m. to 4 p.m. and recorded trials using a video camera (Sony Handycam HDR-CX405). We fed fish in the morning before experiments on the same day. After performing the behavioral trial, each experimental fish was returned to its individual cup and then to the fish colony each day. We used size-matched fish in the experiment, both being experimentally naïve and from different lineages (DAN and H9, respectively). We recorded fish mass (g) and total length (cm) 24 h before experimental trials to size-match fish. The focal fish had an average mass of 0.074 g (± 0.023 g, SD) and an average length of 1.9 cm (± 0.191 cm, SD). The stimulus fish had a comparable average mass of 0.079 g ( ± 0.018 g, SD) and an average length of 2.0 cm (± 0.191 cm, SD). The absolute difference in mass between focal and stimulus fish ranged from 0 to 0.024 g, with the mean absolute difference of 0.008 ± 0.007 g (mean ± SD, *n* = 32 pairs). The absolute difference in length between focal and stimulus fish ranged from 0 to 0.4 cm, with the mean absolute difference of 0.1 ± 0.1 cm (mean ± SD, *n* = 32 pairs).

#### Activity and aggression levels during social exposure

We performed experiments to examine mangrove rivulus social aggression and activity levels with and without psilocybin treatment. We conducted all trials in glass tanks with 15 ppt synthetic seawater, and all individuals were habituated to the experimental room for 1 h to reduce the effect of transporting fish prior to the behavior assessment. We habituated focal fish (DAN) to opaque square glass treatment chambers (8.0 L × 8.0 W × 8.0 H cm; thickness 0.5 cm) filled with 250 mL of seawater and stimulus fish (H9) to the rectangular glass experimental chambers (10.6 L × 8.5 W × 6.8 H cm; thickness 1 cm) filled with 250 mL of seawater for 1 h. These chambers were kept at 26°C, the same temperature as the fish colony. We used a paired design, where we examined an individual’s behavior with and without psilocybin treatment to establish baseline behavioral responses to the stimulus.

**FIGURE 1:**
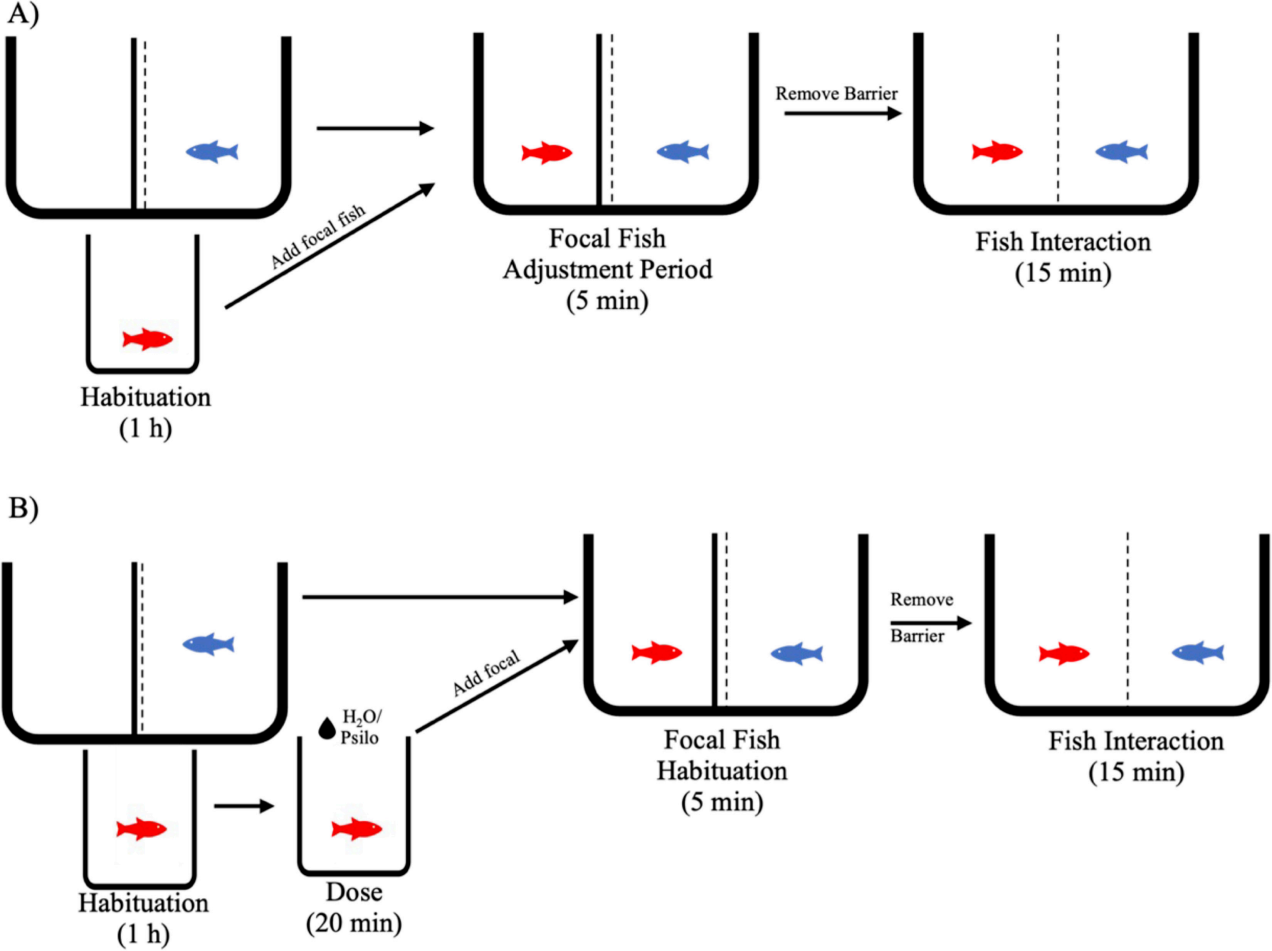
Schematic representation of the experimental protocol indicating: **(A)** time point before treatment, and **(B)** 24 h later (as shown in the figures as “after”) for psilocybin administration via water immersion. Red fish indicate focal fish (DAN) and blue fish indicate stimulus fish (H9).

We exposed focal fish (DAN) to a size-matched stimulus (H9) conspecific for 15 min and 24 h later, treated the same focal fish with a psilocybin dose (20 min) in the square glass chamber before re-introducing it to the experimental chamber with the same stimulus conspecific (Supplementary Figure 1). Following habituation, we added the focal fish to the experimental chamber (rectangular glass chamber with a stimulus fish present) with an opaque cover over the fiberglass mesh barrier allowing the fish to interact visually and chemically (i.e., olfactory) but not physically. After a 5-min adjustment period for the focal fish, we removed the opaque barrier. Both fish were able to interact through the mesh barrier for 15 min (Figure 1). The same protocol occurred 24 h later. We treated the focal fish with either psilocybin or water (control) before adding them to the experimental chamber with the stimulus fish (Figure 1). This paired experiment, with a 24 h interval, allowed us to monitor the behavior of individual fish before and after psilocybin treatment. All our behavioral test data are from psilocybin added to the water. We trialed a food model of delivery with a psilocybin-infused agar worm, but did not include any behavioral data, as the LC-MS analysis revealed that the fish did not take up psilocybin with this method. We feed our lab populations of mangrove rivulus blood worms; thus, we infused an agar “blood worm” with psilocybin (see Supplementary Figure 2).

We manually recorded eight behavioral endpoints:(i) time spent moving (activity), (ii) frequency of head-on and (iii) lateral displays, (iv) time spent in head-on and (v) lateral displays, (vi) frequency of mesh barrier zone entries, (vii) time spent in mesh barrier zone, (viii) frequency of swimming bursts and (ix) time spent interacting (Melanson et al., 2023). The time spent moving is a measure of the activity level of individual fish in their respective halves of the tank. We defined swimming bursts as rapid darts toward the stimulus fish at the mesh barrier; these are a measure of aggressive attack. The mesh barrier zone was defined as the area within one body length of the fish in proximity to the mesh barrier and served as a measurement of interest for the stimulus fish and was not exclusive to aggressive acts. Time spent interacting was a measure of overall interaction time, including, but not limited to, aggressive acts. The other five behavioral endpoints are signs of aggression. We measured all behaviors for the focal fish (DAN) and measured time spent moving and frequency of swimming bursts for the stimulus fish (H9).

### Psilocybin and psilocin concentration – LC-MS

#### Sample collection

We exposed mangrove rivulus fish from the H11 lineage to varying doses of psilocybin to quantify whole-body concentrations and absorption of psilocybin and its active metabolite, psilocin. We used a different fish lineage for these analytical measurements because of availability of fish of the appropriate size and life stage. We recognize that there are genetic differences among lineages, with known differences in metabolism and behavior (Martin et al., 2021; Kelley et al., 2016). Thus, we cannot rule out that psilocybin uptake and absorption would be the same between lineages; however, for our objectives, we reasoned that any differences would be negligible. We exposed fish to psilocybin via water immersion for 20 min at concentrations of 3,000, 6,000, or 12,000 μg/L. We increased the administered concentrations of psilocybin in this analysis compared to the behavioral measurements because we wanted to determine the dose range for whole-body psilocybin and psilocin concentration, as the behavioral trials determined 3,000 μg/L was the lower effective limit. We divided the fish into fasted (48 h) and non-fasted (fed the morning of the experiment) groups to assess the impact of fasting on psilocybin absorption. Immediately after exposure, we euthanized the fish by rapid chilling via ice bath immersion (2°C–4°C) and stored the whole-body samples at −80°C. We later transported the samples in dry ice to Université de Moncton (Moncton, New Brunswick) for extraction and analysis. We also measured psilocybin and psilocin levels in fish receiving psilocybin via an oral dose via an agar blood worm (see Supplementary materials).

#### Calibration standards

We prepared an internal standard solution containing Psilocin D_10_ internal standard (Cerilliant P-099, Millipore Sigma, St Louis, MO, United States) at 30 ng/mL in 0.1% (v/v) formic acid and kept on ice for addition to all calibration standards and fish samples. We prepared an analyte stock of psilocybin and psilocin (Cerilliant P-097 and P-098, Millipore Sigma, St Louis, MO, United States) at 1 μg/mL of each in 0.1% v/v formic acid. This stock was diluted with 0.1% formic acid to produce a 120 ng/mL mixed analyte solution, which serves as our highest calibration curve concentration. Calibration standards ranging from 0.47 to 120 ng/mL, along with a blank, were created by serial dilutions of the 120 ng/mL stock. Each standard was processed identically to samples by combining 60 μL of standard with 300 μL of 80% methanol + 0.1% formic acid and 150 μL of internal standard solution prior to vacuum centrifugation.

#### Sample extraction

We removed the frozen samples from −80°C storage and weighed each fish in a 2 mL Eppendorf tube. To homogenize the tissue, we added 80% v/v methanol in a volume equal to five times the fish’s weight. We homogenized the samples using a probe sonicator (QSonica Q55, Newton, CT, United States) and kept the tissue on ice to minimize the analyte degradation. Psilocin-D10 internal standard was added to all calibration standards and samples as the internal standard for both psilocybin and psilocin. We briefly vortexed the samples and let them rest on ice for at least 5 min. We then centrifuged the tubes at 15,000 rpm for 5 min at 4°C to pellet debris. We carefully transferred the supernatant, which contained psilocybin and psilocin, into clean tubes and used a vacuum centrifuge (Thermo Electron Corporation Savant ISS110 Speed Vac Concentrator, Waltham, MA, United States) to concentrate the samples. Finally, we transferred the concentrated extracts into HPLC vial inserts for LC-MS analysis.

#### LC-MS analysis

We performed quantitative analysis of psilocin and psilocybin using an Agilent Ultivo Triple Quadrupole LC/MS system (Agilent Technologies, Santa Clara, CA, United States). Chromatographic separation was achieved on a Raptor FluoroPhenyl column (100 × 2.1 mm, 5 μm; Restek, Bellefonte, PA, United States) at 40°C. The mobile phases consisted of (A) water containing 0.1% v/v formic acid and (B) methanol containing 0.1% v/v formic acid. The flow rate was maintained at 0.70 mL min^–1^. The gradient was held at 100% A for 2.0 min, then ramped linearly to 100% B at 7.0 min, followed by a return to 100% A at 7.01 min and re-equilibration to initial conditions prior to the next injection. The injection volume was 4 μL, and multiple-reaction monitoring (MRM) was used for detection. The monitored transitions and MS parameters were as shown in Supplementary Table 1. Calibration curves were generated by plotting the peak area ratio (analyte/psilocin-D10) versus nominal analyte concentration and fitted using weighted (1/x) linear regression. Quantification was based on peak areas from the primary MRM transition for each compound, while secondary transitions were monitored for identity confirmation.

### Statistical analysis

We performed all statistical analyses using R statistical software and R Studio (version 4.3.1; R Core Team, 2023; Posit team, 2024) with a critical alpha value (α) of 0.05. All data visualization was completed in GraphPad Prism (version 10.0.2).

We used linear and generalized mixed-effects models to assess the effects of psilocybin on fish behavior. This approach was chosen to account for repeated behavioral measurements on the same individual over time. For all models, we included a random intercept of fish identification to account for individual variability. We examined the effect of psilocybin on social behavior by modeling a response variable (e.g., time spent moving) with fixed effects of treatment (control and psilocybin) and time (before and after treatment), along with their interaction. We applied a Gaussian distribution to our continuous time-based data (e.g., time spent moving) and a Poisson distribution with a log-link function to our count data (e.g., frequency of swimming bursts). Count data models were tested for overdispersion and were not over dispersed. Statistical analyses were performed on the raw data to account for baseline individual variation among fish; we also conducted statistical analysis on the difference in behavior before and after psilocybin exposure (Δ = After–Before).

We used *t*-tests to compare differences between control and psilocybin treatment. Data were evaluated for normality using the Shapiro-Wilk test and for homogeneity of variance using the Levene’s test. For normally distributed data with equal variances, a student’s *t*-test was conducted, if the homogeneity of variance assumption was not supported, a Welch’s *t*-test was conducted. For non-normally distributed data, a Wilcoxon signed rank test was conducted. We conducted an effect size test on statistically significant behaviors.

All models were fitted, validated and the statistical summaries were generated using the easystats package (Lüdecke et al., 2022), including the lme4 (Bates et al., 2015), performance (Lüdecke et al., 2021), and parameters (Lüdecke et al., 2020) R packages. For statistically significant main effects or interactions, we conducted post hoc comparisons using the emmeans package (Lenth, 2023) to determine specific group differences. We conducted the Levene’s homogeneity of variance using the car package (Fox and Weisberg, 2019) and the effect size package (Torchiano, 2020). Significance threshold was α = 0.05.

## Results

### Behavioral effects of psilocybin following water immersion

**FIGURE 2:**
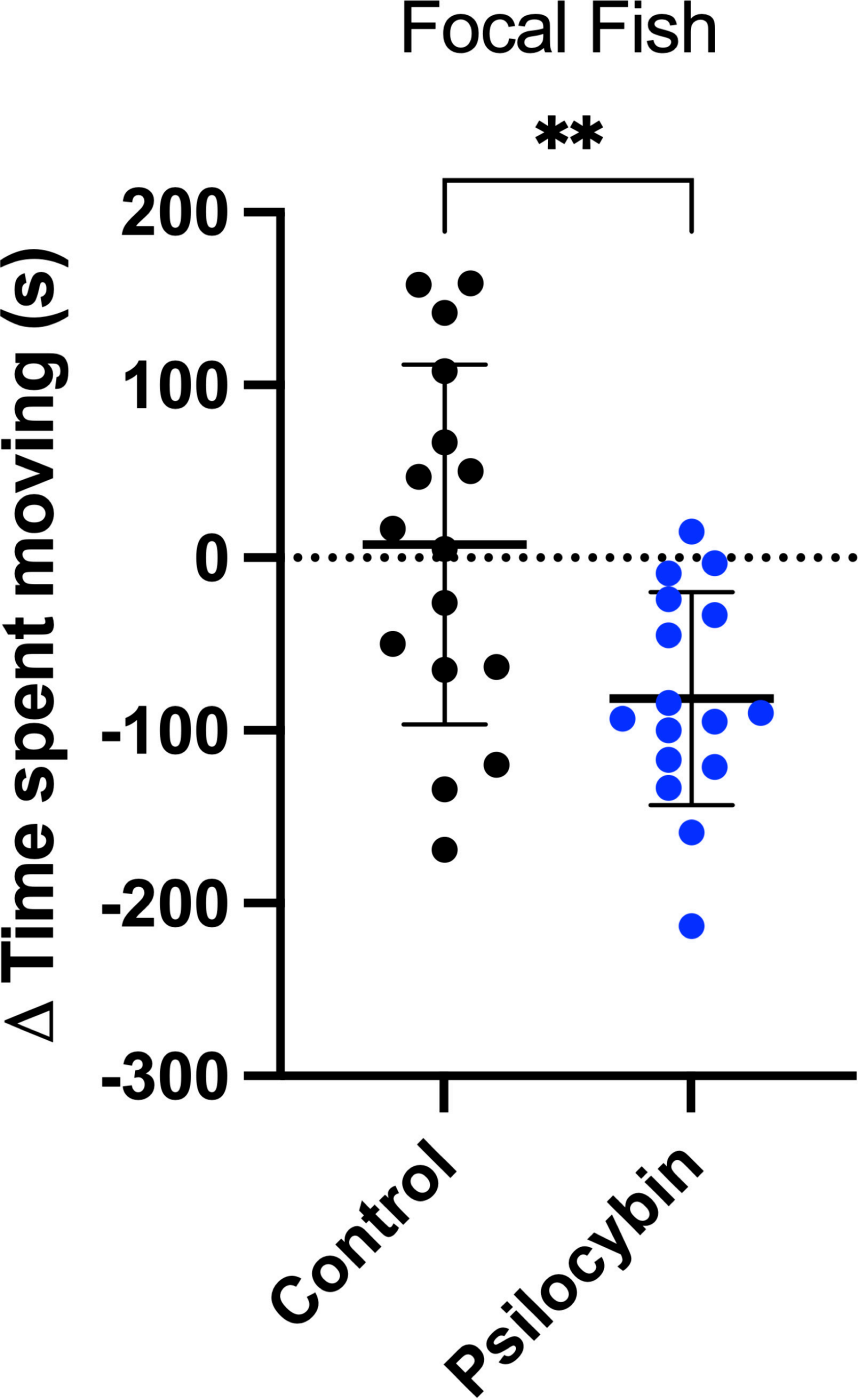
Change in time spent moving (s) of focal *K. marmoratus* following exposure to control and psilocybin treatment via water immersion. Fish were exposed to size-matched stimulus fish for 15 min (before) and 24 h later, focal fish were treated with a control (black) or a psilocybin dose (blue) of 3,000 μ g/L (after). Data points represent the delta change (Δ = After–Before) for each individual (*n* = 16 per treatment; α< 0.05). Asterisks represent the level of significance (**p ≤ 0.01).

We examined the effect of treatment (control and psilocybin) and time (before and after treatment) on eight distinct behavioral endpoints. We observed a significant interaction between psilocybin treatment and time on the time spent moving behavior (*p* = 0.006), suggesting that psilocybin’s effect varies over time. Specifically, fish treated with a water-borne psilocybin dose of 3,000 μg/L significantly decreased their time spent moving from before to after treatment (*p* < 0.001), while no such difference was observed in the control group (Table 1A; *p* = 0.716). Additionally, psilocybin treatment significantly reduced time spent moving compared to the control group on day two (“after”) (Table 1A; *p* = 0.011). We measured the difference between control and psilocybin for each individual to show the change in behavior with treatment. The psilocybin group moved significantly less than the control group, indicating a large treatment effect [Figure 2; *t*(24.4) = 3.0, *p* = 0.007, *d* = 1.04].

**TABLE 1A:** Comparison of treatment effects and time-related differences in control and psilocybin treated groups from activity and aggression levels during social exposure experiments represented in Figures 2–5 (*n* = 16 per treatment).

Behaviour	Linear mixed model	*P*-value
	Comparison	
Time spent moving (s)	Control before–control after	0.716
	Psilocybin before–psilocybin after	**<0.001**
	Control after–psilocybin after	**0.011**
	Control before–psilocybin before	0.397
Frequency of swimming bursts	Control before–control after	0.318
	Psilocybin before–psilocybin after	**<0.001**
	Control after–psilocybin after	**0.007**
	Control before–psilocybin before	0.879
Frequency of mesh barrier zone entries	Control before–control after	0.139
	Psilocybin before–psilocybin after	**<0.001**
	Control after–psilocybin after	0.928
	Control before–psilocybin before	0.115
Time spent in mesh barrier zone (s)	Control before–control after	**0.032**
	Psilocybin before–psilocybin after	**0.021**
	Control after–psilocybin after	0.955
	Control before–psilocybin before	0.8559
Frequency of swimming bursts (stimulus fish)	Control before–control after	**0.003**
	Psilocybin before–psilocybin after	**<0.001**
	Control after–psilocybin after	0.739
	Control before–psilocybin before	0.891

Bold indicates significance (α < 0.05).

**FIGURE 3:**
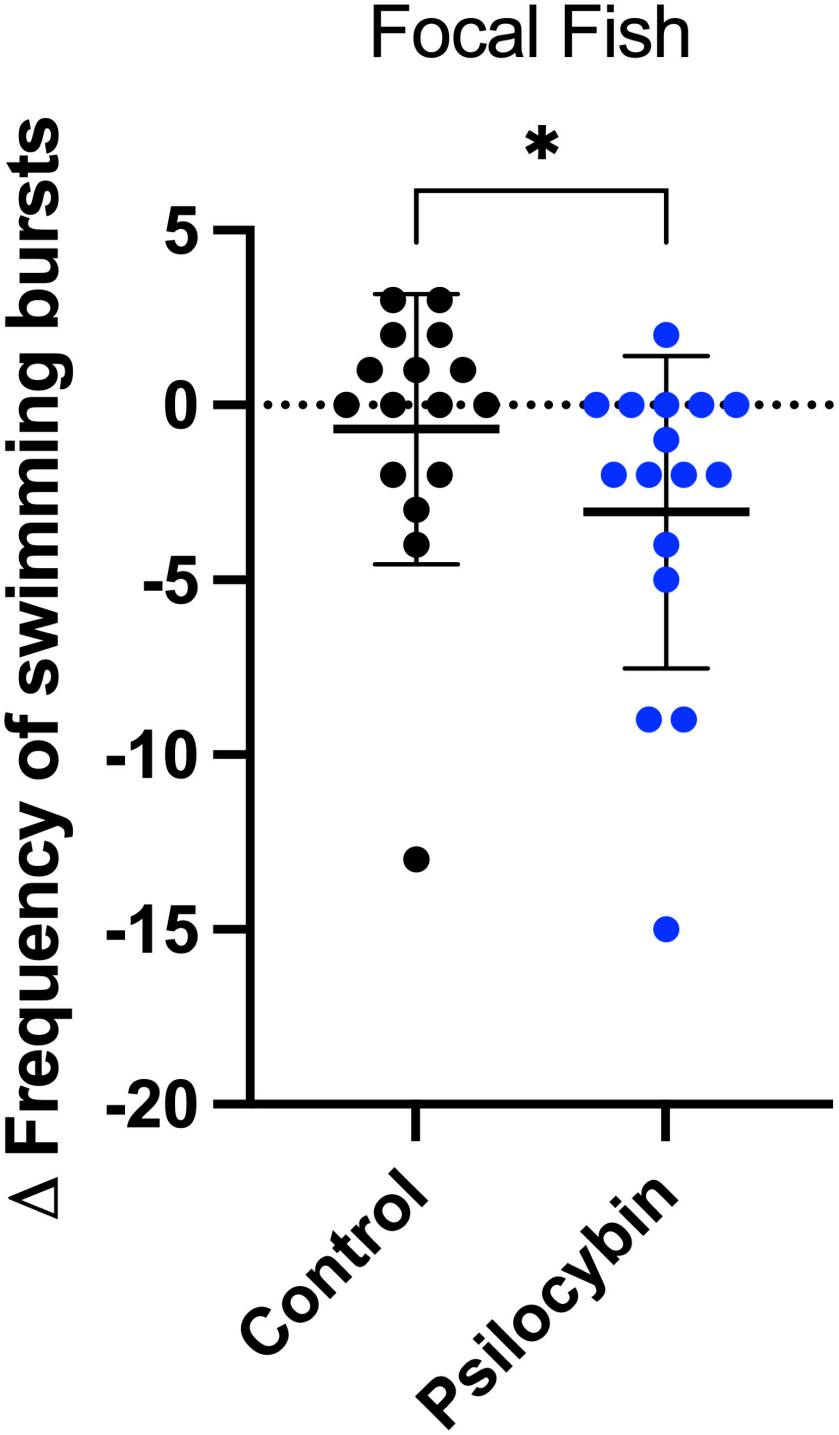
Change in frequency of swimming bursts of focal *K. marmoratus* following exposure to control and psilocybin treatment via water immersion. Fish were exposed to size-matched stimulus fish for 15 min (before) and 24 h later, focal fish were treated with a control (black) or psilocybin dose (blue) of 3,000 μ g/L (after). Data points represent the delta change (Δ = After–Before) for each individual (*n* = 16 per treatment; α < 0.05). Asterisks represent the level of significance (*p ≤ 0.05).

We measured the frequency of swimming bursts as rapid bursts directed toward the stimulus fish on the other side of the mesh barrier. We observed a significant interaction between psilocybin treatment and time on the frequency of swimming bursts (*p* < 0.001), an indicator of aggression towards conspecifics. We observed a significant decrease in swimming burst frequency with psilocybin treatment, with no difference in the control group (Table 1A; psilocybin, *p* < 0.001; control, *p* = 0.318). When comparing day two (“after”) conditions, the psilocybin treated fish exhibited a significantly lower frequency of swimming bursts than the control fish (Table 1A; *p* = 0.007). We also measured other common aggressive behaviors, including biting attempts and mouth tracing by the focal fish towards the stimulus fish within the interaction zone. However, these actions were infrequent, with the majority never occurring at all, regardless of psilocybin treatment. Considering the change in frequency of swimming bursts between control and psilocybin, psilocybin reduced the frequency of swimming bursts compared to control representing a medium effect size (Figure 3; *W* = 182.5, *p* = 0.039, *d* = 0.57).

**FIGURE 4:**
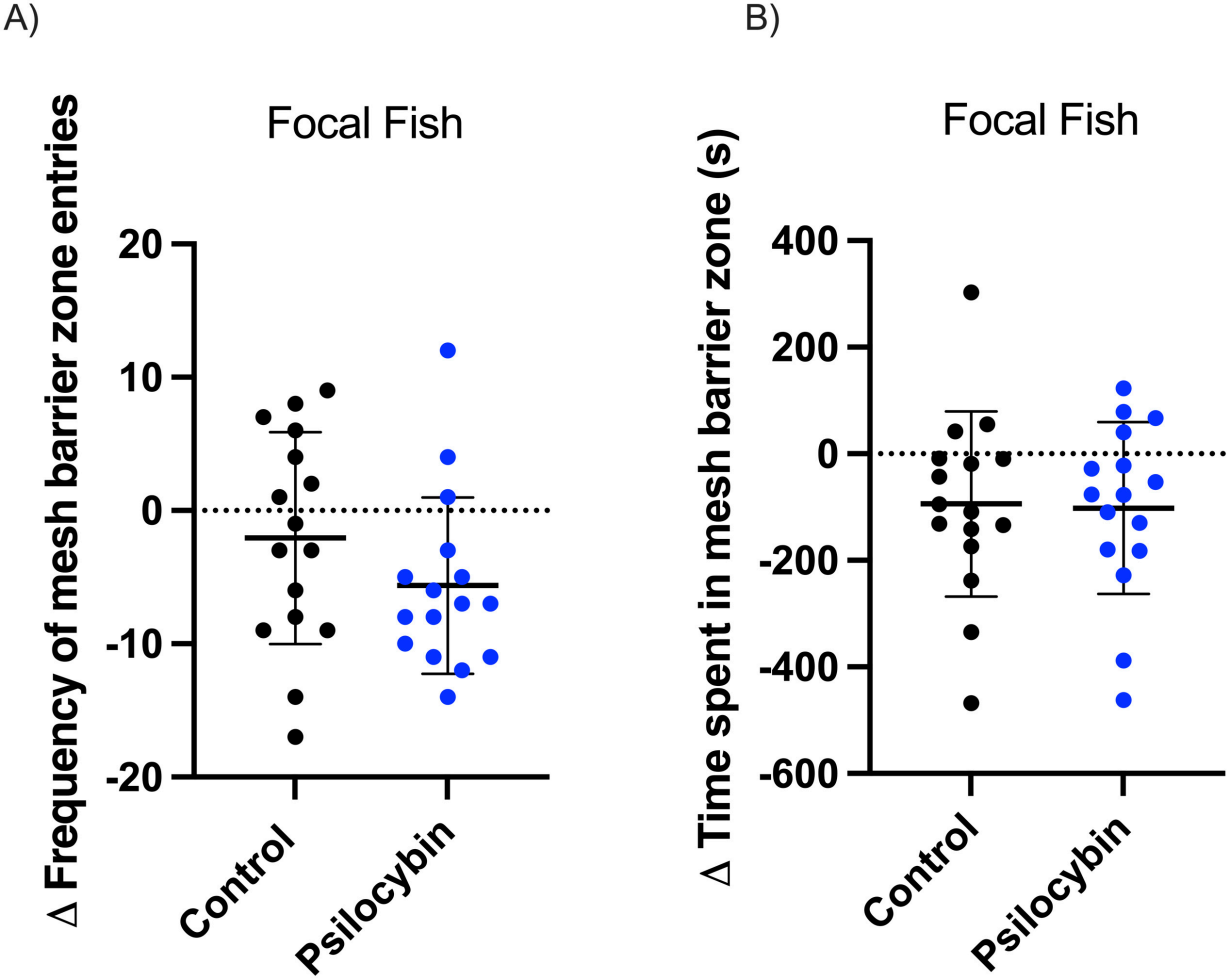
Change in frequency (A) and time (B) of mesh barrier zone entries (within 1 body length from mesh) of focal *K. marmoratus* following exposure to control and psilocybin treatment via water immersion. Fish were exposed to size-matched stimulus fish for 15 min (before) and 24 h later, focal fish were treated with a control (black) or psilocybin dose (blue) of 3,000 μ g/L (after). Data points represent the delta change (Δ = After–Before) for each individual (*n* = 16 per treatment; α< 0.05). No significant differences were detected between groups (*p* > 0.05).

Psilocybin treatment also led to a significant decrease in the frequency of fish entering the zone near the mesh barrier/divider from before to after treatment (*p* < 0.001) compared to control (Table 1A; *p* = 0.139). This behavioral endpoint is a measure of the focal fish’s interest in the stimulus fish. In contrast, the time spent in the mesh barrier zone did not differ between control and psilocybin treatment, but both groups significantly decreased over time (Table 1A; *p* = 0.032, *p* = 0.021, respectively). We did not observe significant differences in the change in frequency of fish entering the zone near the mesh barrier for control and psilocybin [Figure 4A; *t*(30) = 1.4, *p* = 0.1787]. Similarly, no significant differences were observed in the change in time spent in the mesh barrier zone [Figure 4B; *t*(30) = 0.1, *p* = 0.8991].

Psilocybin did not appear to influence the frequency of head-on displays. A head-on display is measured when the focal fish is in a head-on position with the stimulus fish at the mesh barrier. We observed a significant reduction over time (from before to after treatment) in the frequency of head-on displays in both control and psilocybin-treated fish (Table 1B). Similarly, time spent in a head-on display was significantly reduced in both the control and psilocybin treated fish (Table 1B). A lateral display behavior indicates the focal fish “sizing up” the stimulus fish. Time significantly decreased the frequency of lateral displays regardless of psilocybin treatment (Table 1B). However, we observed a more dramatic decline in lateral displays in psilocybin-treated fish. The time spent in a lateral display also significantly decreased with time in both control and psilocybin-treated fish (Table 1B). We observed a significant decrease in time spent interacting in both control and psilocybin-treated fish (Table 1B).

**TABLE 1B:** Comparison of treatment effects and time-related differences in control and psilocybin treated groups from activity and aggression levels during social exposure experiments (*n* = 16 per treatment).

Behaviour	Linear mixed model	*P*-value
	Comparison	
Frequency of head on displays	Control before–control after	**<0.001**
	Psilocybin before–psilocybin after	**<0.001**
	Control after–psilocybin after	0.453
	Control before–psilocybin before	0.078
Time spent in head on displays (s)	Control before–control after	**0.005**
	Psilocybin before–psilocybin after	**<0.001**
	Control after–psilocybin after	0.728
	Control before–psilocybin before	0.068
Frequency of lateral displays	Control before–control after	**0.002**
	Psilocybin before–psilocybin after	**<0.001**
	Control after–psilocybin after	0.185
	Control before–psilocybin before	0.938
Time spent in lateral display (s)	Control before–control after	0.057
	Psilocybin before–psilocybin after	**0.050**
	Control after–psilocybin after	0.375
	Control before–psilocybin before	0.407
Time spent interacting (s)	Control before–control after	**<0.001**
	Psilocybin before–psilocybin after	**0.005**
	Control after–psilocybin after	0.556
	Control before–psilocybin before	0.508

Bold indicates significance (α < 0.05).

**FIGURE 5:**
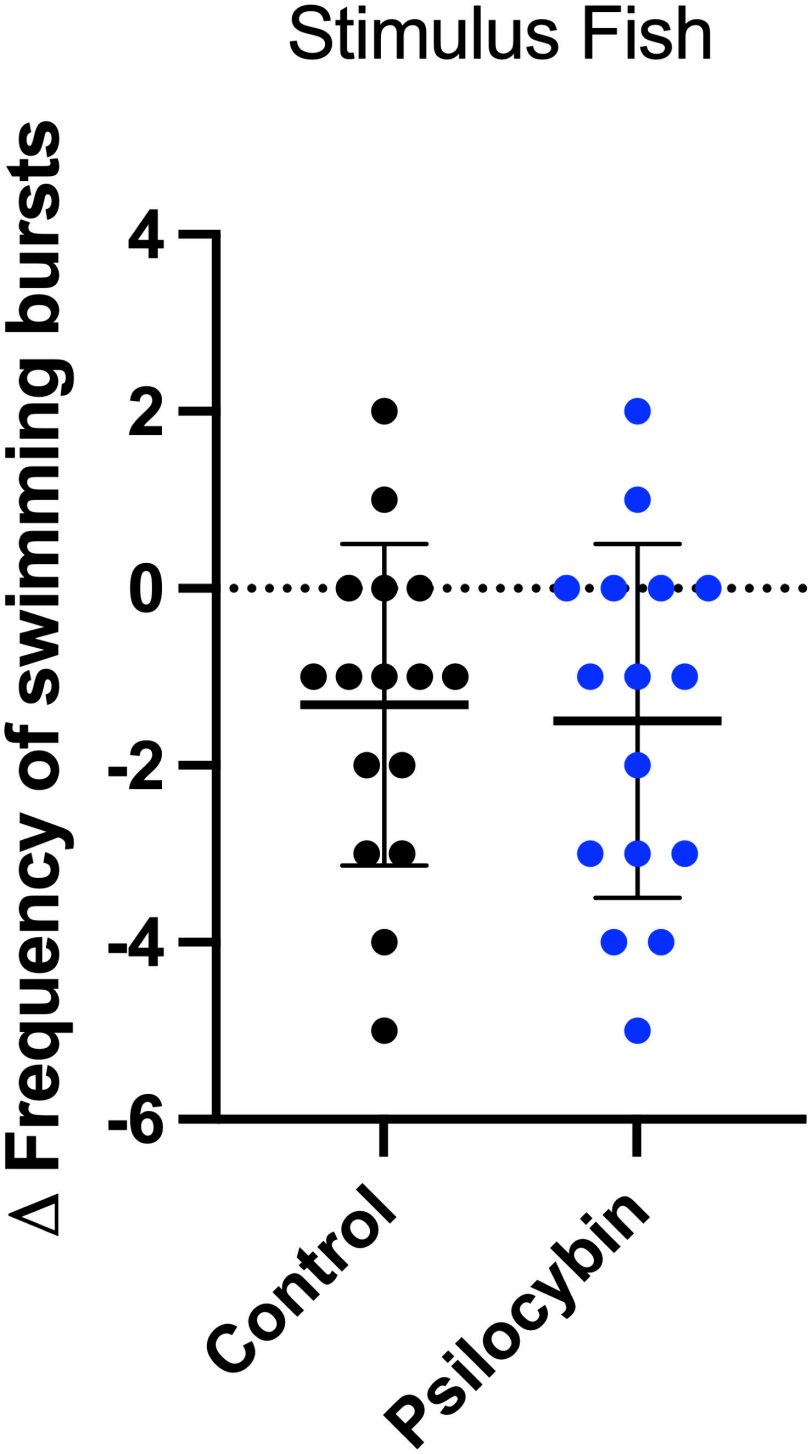
Change in frequency of swimming bursts of *K. marmoratus* stimulus fish. Fish were exposed to size-matched focal fish for 15 min (day 1) and 24 h later, stimulus fish were re-exposed to treated focal fish (day 2). Data points represent the delta change (Δ = After**–**Before) for each individual (*n* = 16 per treatment; α < 0.05). No significant differences were detected between groups (*p* > 0.05).

We were also interested in the stimulus fish’s behavior, as it is known to affect the focal fish’s behavior (Earley et al., 2012; Edenbrow and Croft, 2012a,b). We measured the frequency of swimming bursts and the time spent moving (s) of the stimulus fish, because these key behaviors were where we discovered differences in the focal fish’s behavior with psilocybin treatment. The stimulus fish showed a significant decrease in the frequency of swimming bursts over time from day 1 (before focal fish treatment) to day 2 (after focal fish treatment) regardless of psilocybin treatment (Table 1A; control, *p* = 0.0027, psilocybin, *p* = 0.0007; respectively). We did not observe a significant change over time in their time spent moving (Supplementary Figure 4). We did not observe significant differences in the change in frequency of swimming bursts in the stimulus fish for control and psilocybin [Figure 5; *t*(30) = 2.8, *p* = 0.7832].

### Psilocybin and psilocin concentration – LC-MS

**FIGURE 6:**
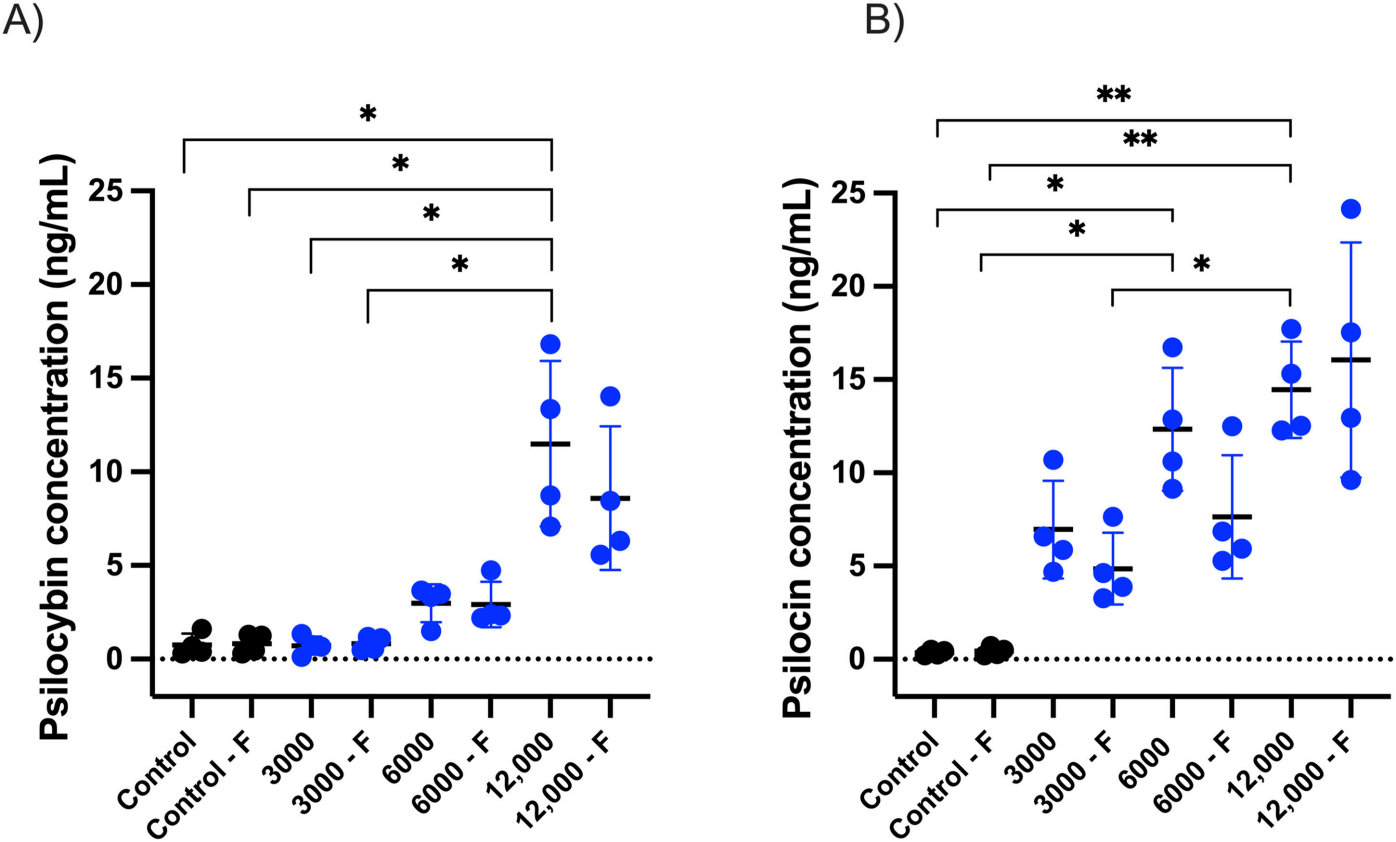
Whole-body concentrations (ng/mL) of psilocybin **(A)** and psilocin **(B)** in focal *K. marmoratus* following exposure to psilocybin 3,000, 6,000, or 12,000 μg/L via water immersion (20 min) with or without fasting. Circles represent individuals (*n* = 4 per treatment) with black circles representing control fish and blue representing psilocybin treated fish. Fasted fish are represented with the letter F. Asterisks represent the level of significance (*p ≤ 0.05, **p ≤ 0.01).

As psilocybin is rapidly converted to its active form, psilocin, we measured both psilocybin and psilocin concentrations in fish exposed to psilocybin via water immersion (3,000, 6,000, and 12,000 μg/L for 20 min) or oral administration (Supplementary Figure 3). The water immersion groups included both fasted and non-fasted fish. We observed that psilocybin and psilocin levels increased with higher psilocybin doses via water immersion, and there were no significant differences between fasted vs. non-fasted fish (Figures 6A, B).

## Discussion

Fish models offer significant advantages for neuropharmacological and behavioral studies due to their high physiological and genetic homology to humans (Shin and Fishman, 2002; Adeyinka et al., 2025), facilitating more robust results with potential implications for human use. Notably, psilocybin’s effects on social behavior, regardless of animal model, remain largely undescribed. Capitalizing on the utility of the mangrove rivulus fish as a model for investigating social behavior/aggression (Earley et al., 2012), we predicted that a single dose of psilocybin would reduce aggression and activity in mangrove rivulus in a dyadic pair. Our findings demonstrate that waterborne psilocybin at 3,000 μg/L significantly decreased activity levels and aggressive swimming bursts, suggesting a calming effect. We also observed time-dependent changes in behavior among control fish, underscoring the importance of accounting for baseline behavioral variation in individual fish when studying the effects of pharmacological agents.

As predicted, waterborne psilocybin treatment led to significant reductions in time spent moving, consistent with psilocybin’s known sedative and calming effects. When mangrove rivulus focal fish encounter a conspecific, they tend to increase their activity (Yin-Liao et al., 2019; Melanson et al., 2023). Therefore, in our study, we paired our focal fish with a stimulus (conspecific) fish to elicit a response, as they are naturally aggressive fish, and to determine if psilocybin would reduce their activity and aggression. Studies in larval zebrafish have reported variable outcomes with psilocybin treatment, including both increased swimming (Braun et al., 2024) and no significant change in swimming behaviors (Tombari et al., 2023). These discrepancies may reflect differences in life stage, dosage, or experimental conditions. Regardless, our data suggest that a single dose of psilocybin at 3,000 μg/L administered via water immersion influences the activity levels of adult mangrove rivulus compared to controls.

Research on the putative effects of psilocybin on aggression is important because it provides insight into how psilocybin modulates aggressive behaviors, critical aspects of social interactions and mental health in humans. Investigations into alterations in aggressive behavior in response to psilocybin treatment in animal models are a relatively unexplored area of research, although aggressive behavior has been shown to be inhibited by psilocybin in rodents (Kostowski et al., 1972; Uyeno, 1978). Mangrove rivulus, as a model species, is particularly beneficial to study the potential effects of psilocybin on this behavior, as they are inherently aggressive fish (Hsu and Wolf, 1999). Their aggressive tendencies are modulated by prior fighting situations, where when an individual was previously in a fighting situation with another fish and was successful, they exhibited bolder behavior and became more likely to initiate another fight (Hsu and Wolf, 2001) and losing causes them to retreat when confronted and display shyness (Hsu et al., 2005). Our data indicate that a 3,000 μg/L waterborne dose significantly reduced the frequency of swimming bursts compared with the control group but did not affect other aggressive behaviors. This specific decrease in swimming bursts suggests a trend towards calmer social interactions with waterborne psilocybin treatment, even within a species known for its innate aggressiveness (Hsu and Wolf, 1999).

Social interactions are complex, and it is widely recognized that the behavior of the stimulus fish has an effect on the focal fish’s behavioral response (Bass and Gerlai, 2008; O’Connor et al., 2015; Hotta et al., 2015). For instance, a highly aggressive and active bold personality stimulus fish elicits a different response in the focal fish than a shy passive fish (Pike et al., 2008). Therefore, measuring the stimulus and focal fish behavior is important for assessing drug effects in this model. Within this social exposure model, the element of time played a significant role as the focal fish did not display consistent reactions over time, likely due to familiarity with the stimulus fish. We prioritized a paired design to track individual behavior over time, considering the inherent variability within a single isogenic lineage of these fish (Edenbrow and Croft, 2011, 2012b). Thus, any psilocybin effect had to overcome not only individual variability but also the influence of repeated social exposure, as observed in the control group. Regardless of the effect of time spent with the stimulus fish, we continued to observe psilocybin’s influence on certain key behaviors. Furthermore, our preliminary tests found that changing stimulus fish each day did not alter behavior compared to keeping the same stimulus fish, suggesting that stimulus identity did not likely make a difference in post-treatment behavioral responses.

Our lab populations of mangrove rivulus fish are isogenic, allowing us to control for genetic variability (Kelley et al., 2016; Tatarenkov et al., 2015). We used one isogenic lineage as the focal fish in the behavioral trials and observed considerable individual variability in behavior, likely due to differences in epigenetic, physiological, and personality traits as has been previously shown in mangrove rivulus lineages (e.g., Dundas et al., 2026; Mathiron and Silvestre, 2025; Mathiron et al., 2023; McCain et al., 2020; Edenbrow and Croft, 2011, 2012b). Indeed, this variability persisted in the stimulus fish, from a different lineage. Thus, our paired experimental design takes into account this individual variability to give us baseline behavioral data.

Our whole-body LC-MS analysis indicated that psilocybin and psilocin concentrations were generally low compared to the concentration administered. Dose comparisons between aquatic animals and humans are inherently difficult, particularly when equating the concentration in a water bath (3,000 μg/L) to a single internal dose. However, the resulting low tissue concentration (∼7 ng/mL) is similar to the peak plasma psilocin concentration (11 ng/mL) reported in humans following a low to moderate 15 mg dose (Holze et al., 2023; Fang et al., 2024). The mangrove rivulus is an amphibious fish that exhibits cutaneous respiration during emersion from the water using its richly vascularized skin for gas exchange in addition to its gills (Wright, 2012). Given that these fish use both their gills and skin for respiration, they are likely adept at absorption of waterborne chemicals. That said, there are factors that may reduce the uptake of psilocybin. In this study, mangrove rivulus were isotonic to their brackish environment (15 ppt) and therefore did not drink water, as fish do when hypo-osmotic relative to their environment (Greenwell et al., 2003). Furthermore, the phosphate group on psilocybin may impair passive diffusion across gills and skin, limiting uptake (Thomann et al., 2024; Wiemer, 2020). However, the low measured concentration observed with waterborne psilocybin is not a universal characteristic of chemical uptake in fish. For example, Abramson et al. (1963) found that while waterborne psilocybin exposure did not show a comparable effect to injection, waterborne LSD yielded similar results to injection, suggesting compound-specific differences in efficacy. Surprisingly, we detected trace concentrations of psilocybin and psilocin in control fish. We speculate that these low levels were due to post-mortem contamination during the psilocybin and psilocin extraction process. These trace contamination values remained well below those measured in the waterborne psilocybin treated fish, confirming that the treatment group received and retained a biologically effective dose.

Psilocybin has a similar structure to serotonin, explaining its high affinity for serotonin receptors and, therefore, mimicking its effects (de Veen et al., 2017). This structural similarity allows psilocybin to bind to serotonin receptors effectively, thereby modulating stress responses and aggression. The known role of serotonin in modulating responses to stressors, including conspecific aggression (Clotfelter et al., 2007), is consistent with the behavioral changes observed in our study. Future studies should focus on investigating serotonin levels and the specific receptor mechanisms in the mangrove rivulus to explain the physiological bases of these observed behavioral effects.

Overall, our study demonstrated that an acute waterborne dose of 3,000 μg/L of psilocybin reduced aggression and activity levels in adult mangrove rivulus. To our knowledge, this is the first study to demonstrate that psilocybin reduces aggression in any animal model. Our findings reveal calming behavioral changes in adult fish, evidenced by decreased swimming bursts and time spent moving, in response to water-borne psilocybin exposure. These results provide valuable insight into how a naturally aggressive species can be affected by psilocybin, reducing both aggression and activity during interactions with a conspecific. Our data contribute to our understanding of psilocybin’s impact on the behavior and physiology of this unique hermaphroditic fish species, with broader implications for the study of psychoactive substances and behavioral neuroscience in other species. Future research in this area will offer essential insights, potentially guiding the therapeutic use of psilocybin in clinical settings for humans.

## Data Availability

The original contributions presented in the study are publicly available. This data can be found in Borealis: https://doi.org/10.5683/SP3/J3QF7K.
