# Supplementary material for: The magic of mushrooms: psilocybin influences behavior in the mangrove rivulus fish, Kryptolebias marmoratus
Source: Front Behav Neurosci. 2026 May 7;20:1767175. doi: 10.3389/fnbeh.2026.1767175 (PMC13190583; doi:10.3389/fnbeh.2026.1767175)
Supplement: Supplementary file 1 [file Data_Sheet_1.docx]

Supplementary Material

**Methods and Materials**

**Behavioural effects of psilocybin following administration via food**

## Pharmacological Treatment

We administered psilocybin to each fish through oral ingestion in an agar worm form, simulating a blood worm meal, to investigate the effect of an oral dose of psilocybin to assess its feasibility and consistency compared to water immersion. We created this new protocol where the active ingredient is incorporated into an agar worm vehicle. We prepared the worms in two batches to ensure freshness: one before the initial exposure and a second batch halfway through the experimental day. We created a custom silicone mold using a 3D printer and used silicone to create the mold that was used to make agar worm forms using agar mixed with bloodworm water and red and blue food colouring (Twinkle Baker Food Colour purchased from Giant Tiger, NS, CA) to mimic the real bloodworms that are part of their weekly diet. To make the bloodworm water, we placed frozen bloodworms into Reverse Osmosis water and left them to soak in water for 30 min to gain the flavouring of the bloodworms but minimizing the nutritional value. We made the control agar worms using agar mixed with bloodworm water and food colouring, while the psilocybin treated agar worms were made using agar mixed with bloodworm water, food colouring, and psilocybin at a dose of 3000 µg/L, based on previous water-immersion experiments. Each focal fish (H11 lineage) was placed in its experimental chamber and given either a control or a psilocybin-treated agar worm. Due to limited stock of the original lineage used in psilocybin water immersion experiments as focal fish, we used a different established lineage of mangrove rivulus for these experiments.

## Experimental Protocol

We analyzed fish behaviour through blinded video analysis. As in water immersion dosing experiments, we conducted all experiments from 10 am to 4 pm and recorded trials using a video camera (Logitech 4K Pro Webcam, San Jose, CA, USA). All fish were fasted for 24 h prior to day 1 of the experiments and fasted for 48 h before day 2 of the trials. Preliminary experiments (data not reported) were conducted to determine the length of fasting to ensure fish would be motivated to consume the agar worm consistently. After performing the behavioural trial, each experimental fish was returned to its individual cup and then to the fish colony. We used size-matched fish in this experiment with both the focal and stimulus fish being experimentally naïve and from different lineages (H11 and H9, respectively). We recorded fish mass (g) and total length (cm) 24 h before experimental trials to size match fish. The focal fish had an average mass of 0.072 g (± 0.014 g, SD) and an average length of 2.0 cm (± 0.1 cm, SD). The stimulus fish had a comparable average mass of 0.075 g (± 0.016 g, SD) and an average length of 2.0 cm (± 0.2 cm, SD). The absolute difference in mass between focal and stimulus fish ranged from 0 g to 0.025 g, with the mean absolute difference of 0.005 g ± 0.004 g (mean ± SD, n = 64 pairs). The absolute difference in length between focal and stimulus fish ranged from 0 cm to 0.4 cm, with the mean absolute difference of 0.1 cm ± 0.08 cm (mean ± SD, n = 64 pairs).

### Activity Levels during Social Exposure

We performed these experiments as in water immersion dosing experiments with the following minor modifications. We habituated focal fish to rectangular glass chambers (10.6 L x 8.5 W x 6.8 H cm; thickness 1 cm) filled with 250 mL of synthetic seawater for 1 h, and a size-matched stimulus fish in its respective 120 mL plastic cup with approximately 80 mL of synthetic seawater for habituation. We used a paired design, where we examined an individual’s behaviour with and without psilocybin treatment to establish baseline behavioural responses to the stimulus. Also, we used a different lineage of fish as focal fish because of availability and size of the fish.

We exposed a focal fish (H11) to a size-matched stimulus (H9) conspecific for 15 min and 24 h later, treated the same focal fish with an oral psilocybin dose waiting 15, 30 or 60 min after feeding to begin the trial with the same stimulus conspecific (Fig. S2). Following habituation, we added the stimulus fish to the experimental tank with the focal fish present on the other side (rectangular tank:10.6 L x 8.5 W x 6.8 H cm) with an opaque cover over the fiberglass mesh barrier allowing the fish to interact visually and chemically (i.e., olfactory) but not physically. After a 5-min adjustment period for the stimulus fish, we removed the opaque barrier, and both fish were able to interact through the mesh barrier for 15 min (Fig. S2). The same protocol occurred 24 h later. We gave the focal fish either a psilocybin- or water-infused agar worm (control). We waited either 15, 30, or 60 min after feeding prior to adding the stimulus fish to the experimental tank (Fig. S2). As in the water immersion experiments, this paired experiment and 24 h timing allowed us to monitor the behaviour of individual fish before and after psilocybin treatment (Fig. S1). We manually recorded time spent moving (activity) for the focal fish (H11).

**Psilocybin & Psilocin Concentration – LC-MS**

Sample Collection

We exposed mangrove rivulus fish from the H11 lineage to varying doses of psilocybin to quantify whole-body concentrations and absorption of psilocybin and its active metabolite, psilocin. We exposed fish to psilocybin via oral administration for 30 min at a concentration of 12,000 µg/L. We initially chose 15-, 30-, and 60-min exposure times for the behavioural assessment, with the 30 min duration being chosen for this experiment because it yielded the most distinct behavioural trend, although not statistically significant. This choice was further supported by preliminary LC-MS results at these different exposure times (data not reported). Before oral dosing, we fasted the fish for 48 h to ensure they consumed the psilocybin-infused agar worm. Immediately after exposure, we euthanized the fish by rapid chilling via ice bath immersion (2°C to 4°C) and stored the whole-body samples at -80˚C. We later transported the samples in dry ice to Université de Moncton (Moncton, New Brunswick) for extraction and analysis.


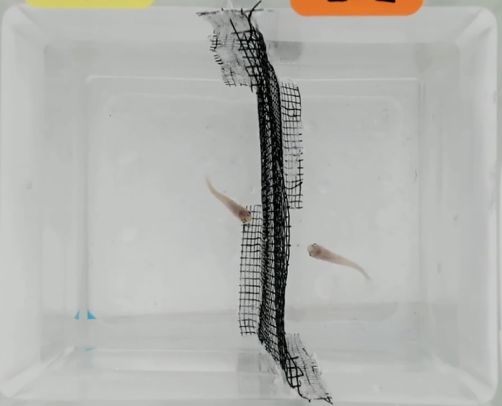
 Figure S1. Photograph of the social exposure testing experimental setup with mesh barrier allowing fish to interact visually and chemically, but not physically.


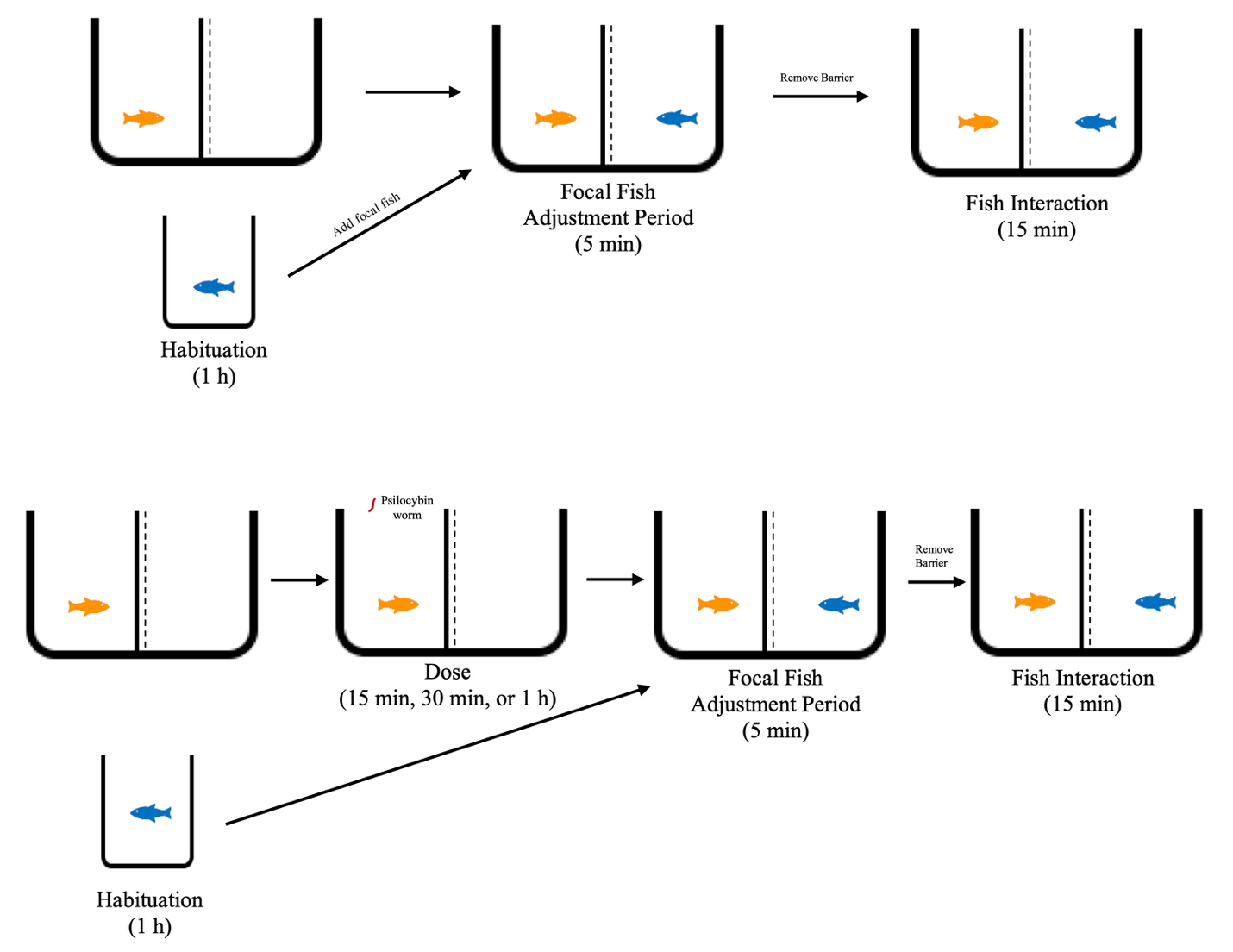


Figure S2. Schematic representation of the experimental protocol, indicating: A) time point before treatment, and B) 24 h later (as shown in the figures as “after”) for psilocybin administration via oral agar worm. Orange fish indicate focal fish (H11) and blue fish indicate stimulus fish (H9).

**Results**

**Psilocybin & Psilocin Concentration – LC-MS**

We measured psilocybin and psilocin concentrations in fish exposed to psilocybin via oral administration at 12,000 µg/L for 30 min. Oral administration did not result in detectable levels of psilocybin, and only trace amounts of psilocin (Fig. S3).

1. B)

**Figure S3. Whole-body concentrations (ng/mL) of psilocybin (A) and psilocin (B) in *Kryptolebias marmoratus* following exposure to psilocybin at 12,000 µg/L via oral administration (30 min).** Circles represent individuals, with black circles representing control fish (n = 5 per treatment) and blue representing psilocybin treated fish (n = 4 per treatment). Asterisks represent the level of significance (**p* $\boldsymbol{\leq}$0.05, ***p* $\boldsymbol{\leq}$0.01**,** ****p* $\boldsymbol{\leq}$0.001**;** no significant differences were observed).

**Behaviour of Stimulus Fish**


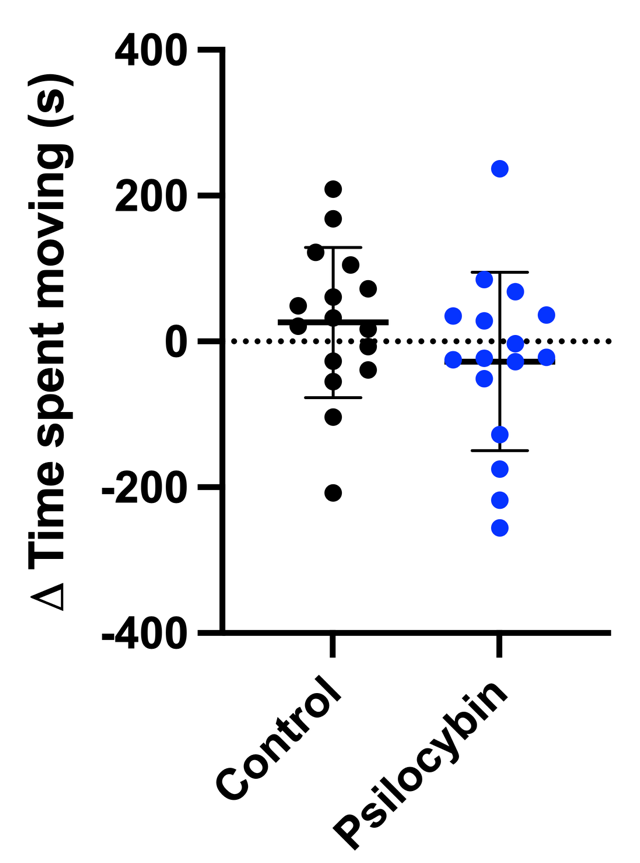
We examined the behaviour of the stimulus fish that were paired with each of the focal fish in both control and psilocybin treatment groups. The stimulus fish showed no change in their time spent moving (s) over time from day 1 (before focal fish treatment) to day 2 (after focal fish treatment) in both groups (control, *p*=0.795, psilocybin, *p*=0.766; respectively). We observed no significant difference in the delta values between control and psilocybin (Fig. S4, *p* = 0.191).

Figure S4. Change in time spent moving (s) of *K. marmoratus* stimulus fish from water immersion experiments. Fish were exposed to size-matched focal fish for 15 min (day 1) and 24 h later, stimulus fish were re-exposed to treated focal fish (day 1). Data points represent the delta change (Delta = After – Before) for each individual (n = 16 per treatment; α < 0.05). Asterisks represent the level of significance (**p* $\boldsymbol{\leq}$0.05, ***p* $\boldsymbol{\leq}$0.01; no significant differences were observed).

Table S1. Multiple reaction monitoring (MRM) transitions and MS parameters for quantification of psilocin and psilocybin, with psilocin-D₁₀ used as the internal standard for both compounds.

| Compound | Precursor (m/z) | Product (m/z) | Dwell (ms) | Fragmentor (V) | Collision Energy (V) |
| --- | --- | --- | --- | --- | --- |
| Psilocin | 205.1 | 160.1 | 100 | 90 | 16 |
| Psilocin | **205.1** | **57.9** | **100** | **90** | **16** |
| Psilocin-D_10_ | 215.2 | 164.1 | 100 | 90 | 16 |
| Psilocin-D_10_ | **215.2** | **66.1** | **100** | **90** | **16** |
| Psilocybin | 285.1 | 240.1 | 100 | 120 | 16 |
| Psilocybin | **285.1** | **58.0** | **100** | **120** | **32** |

Note: *Transitions in **bold** were used for quantification; non-bold transitions were used for confirmation of analyte identity.
